# Supplementary figures and images for: Authorship trends in infectious diseases society of America affiliated journal articles conducted in low-income countries, 1998–2018
Source: PLOS Glob Public Health. 2022 Jun 10;2(6):e0000275. doi: 10.1371/journal.pgph.0000275 (PMC10021251; doi:10.1371/journal.pgph.0000275)

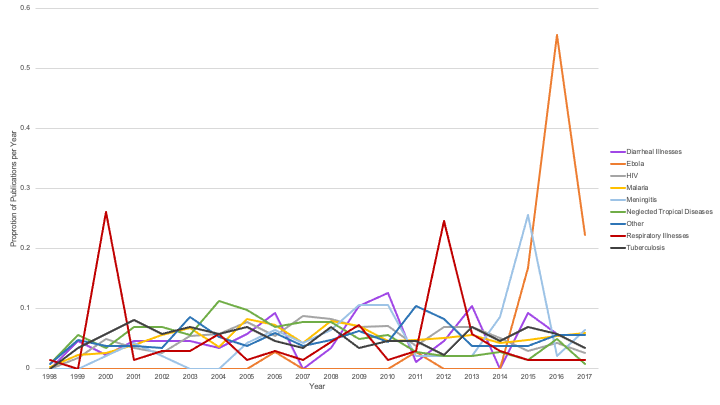

Supplement: S1 Fig — (TIFF) [file pgph.0000275.s001.tiff]
