# Supplementary material for: Authorship trends in infectious diseases society of America affiliated journal articles conducted in low-income countries, 1998–2018
Source: PLOS Glob Public Health. 2022 Jun 10;2(6):e0000275. doi: 10.1371/journal.pgph.0000275 (PMC10021251; doi:10.1371/journal.pgph.0000275)
Supplement: Attachment — Submitted filename: Reviewer Response_FINAL.docx [file pgph.0000275.s003.docx]

February 17, 2022

We have incorporated or responded to all of the suggestions made by the editors and reviewers. Please see below for point-by-point responses. All page numbers refer to the revised manuscript file with tracked changes.

**Journal/Editor Comments**

**Comment 1:** Please amend your Data Availability Statement and indicate where the data may be found (domain name and direct link).

Response: We have added a data file (Supplement 2) which includes the raw and stratified data sets used in the analysis. The Data Availability Statement has been updated to the following:

“The authors confirm that the data supporting the findings of this study are available within the article and its supplementary materials.”

**Comment 2:** Please amend your detailed Financial Disclosure statement. This is published with the article, therefore should be completed in full sentences and contain the exact wording you wish to be published. ii). State the initials, alongside each funding source, of each author to receive each grant. iii). State what role the funders took in the study. If the funders had no role in your study, please state: “The funders had no role in study design, data collection and analysis, decision to publish, or preparation of the manuscript.”

Response: The Financial Disclosures statement has been updated to the following:

“This work was funded by Imagine, Innovate and Impact (I3) Funds from the Emory School of Medicine and through Georgia CTSA NIH award (UL1-TR002378). C.E.M. was supported as a post-doctoral fellow of the Oxford-Johns Hopkins Global Infectious Disease Ethics Collaborative funded by the Wellcome Trust [grant numbers 221719 and 216355]. The funders had no role in study design, data collection and analysis, decision to publish, or preparation of the manuscript.”

**Comment 3:** Please include the Funding Information in the system. This should have the same information of Financial Disclosure statement.

Response: We apologize for the incompleteness of the original submission. The Funding Information in the submission system has been updated to reflect the same text as the Financial Disclosure statement.

**Comment 4** (from revision response dated 2/15/22): Please remove your response to journal requirements from your cover letter and include it with response to reviewers.

Response: This has been updated.

**Comment 5** (from revision response dated 2/15/22): Please provide us with a direct link to the base layer of the map used in Fig 4 and ensure this location is also included in the figure legend.

Page 10, Lines 211-212: “…or last authorship. Maps were created using Plotly Chart Studio (Plotly Technologies Inc, released 2015). Abbreviations…”

Response: We greatly appreciate the attention to appropriate map source citing. Our original figure was created using standard image templating in Microsoft Powerpoint but upon further investigation into the source appears to be copyrighted by Microsoft and the Australian Bureau of Statistics. We have therefore generated a new map for Figure 4 using Plotly Chart Studio (Plotly Technologies Inc, released 2015) using an open source map template. The software is cited in the figure caption. More information about this software is available at https://plotly.com.

**Reviewer 1 Comments**

**Comment 1:** Authors may consider expanding the Introduction and Discussion to include the following relevant/recent publications:

- <https://gh.bmj.com/content/6/1/e003758>
- <https://gh.bmj.com/content/4/5/e001853.abstract>
- <https://europepmc.org/article/med/21144255>

These publications also cite other topic-specific studies of authorship metrics that authors may or may not find relevant.

Response: Thank you for sharing these articles. They are all very relevant to the subject and discussion of our manuscript and we have incorporated them into the Discussion section.

Page 11, Lines 247-248: “…from these areas. While trends in some journals has shown that the vast majority of lead authors were from LICs (22), other analyses…”

Page 12, Lines 256-259: “…where the research took place (20). Large-scale metadata on global health research over the past 20 years indicates that while the proportion of non-HIC lead authors has increased over time, the majority of this trend was secondary to increases in authorship from upper-middle- and lower-middle- income countries, with LICs remaining underrepresented (26).”

Page 15, Lines 326-329: “A similar analysis of academic partnerships within sub-Saharan Africa showed that LIC authorship representation reduced significantly in articles published in collaboration with HICs, in particular when collaborating with high-ranking universities from the United States (39).”

Pages 16, Lines 360-365: “…wider range of journal impact factors. For example, in their analysis of authorship in sub-Saharan Africa, Hedt-Gauthier et al. found that over half (60.6%) of articles published in journals without impact factors had local authorship representation compared to only 4.8% of papers published in journals with impact factors over 10 (39). A similar analysis within infectious disease literature and broader geographic focus would be of interest and relevant to our findings.”

New References:

(22) Plaisant A, Pierard C, Beyers N, Chan-Yeung M, Enarson DA. Authorship affiliations in original articles submitted to the International Journal of Tuberculosis and Lung Disease. Int J Tuberc Lung Dis. 2010;14(12):1650-2.

…

(26) Dimitris MC, Gittings M, King NB. How global is global health research? A large-scale analysis of trends in authorship. BMJ Glob Health. 2021;6(1).

…

(39) Hedt-Gauthier BL, Jeufack HM, Neufeld NH, Alem A, Sauer S, Odhiambo J, et al. Stuck in the middle: a systematic review of authorship in collaborative health research in Africa, 2014-2016. BMJ Glob Health. 2019;4(5):e001853.

**Comment 2:** Authors may consider reframing parts of the Introduction and Discussion from the perspective(s) of LIC researchers, including the barriers LIC researchers may face, and the contribution of HIC-based systems of research/dissemination to those barriers. I think there are a few statements that could be interpreted as shifting the onus on LIC researchers/systems rather than acknowledging the role that HIC researchers/systems may have in perpetuating the inequities that the authors describe (I recognize this is likely not the authors' intended meaning). I have highlighted some specific statements below that the authors may consider rewording.

Response: We really appreciate this point and think it is very important to address. We have incorporated the reviewer’s feedback on these statements in addition to the statements highlighted by the reviewer below.

Page 3, Lines 51-53: “...these are the geographic areas that have historically - and continue - to face significant systematic barriers to achieving equitable academic resources, institutional infrastructure, and economic development compared to high-income countries (HICs).”

Page 3, Lines 54-59: “…the establishing of research priorities, definitions of what constitutes ‘meaningful’ research, and financial support for both primary research and research capacity building are dominated by HIC voices. Combined with the greater academic, institutional, and economic resources at the disposal of HIC investigators these inherent asymmetries in global health and infectious disease research end up perpetuating existing academic inequalities.”

Page 17, Lines 396-397: “…and low-income settings in such a way that does not place additional burden on the LIC partner.”

**Comment 3:** Abstract, page 2, lines 26-27: “…the contribution of LIC investigators as measured by authorship metrics has been poorly studied”. I believe this statement is incorrect (see above publications as a sample of other studies that have investigated authorship metrics); perhaps “…the contribution of LIC investigators as measured by authorship metrics, specifically to infectious diseases research, has not been thoroughly studied” or something similar would be more accurate.

Response: Thank you for pointing this out. This type of research has been expanded upon considerably within the past few years. We have adjusted the text (page 2, lines 26-28) to better reflect the deficiency in the literature in particular to infectious disease publications with phrasing as suggested by the reviewer.

**Comment 4:** Introduction, page 3, lines 47-50: “While the predominant burden of infectious diseases falls within LICs, these are the geographic areas that have historically lacked the resources and infrastructure to generate impactful locally-led research (4, 5). Although there has been substantial interest in developing research capacity within LICs (6-8), significant obstacles still exist.” See above comment re Introduction/Discussion from the perspective(s) of LICs. Authors may consider checking whether the articles they cite find that LICs do not "generate impactful locally-led research" (i.e. is it possible that impactful locally-led research is generated, but LICs experience barriers in publishing this in HIC-based journals with high impact factors?). Similarly, have LICs "lacked resources and infrastructure" or have LICs encountered barriers in obtaining funding/building infrastructure? Who, specifically, has expressed "substantial interest in developing research capacity"; researchers from LICs, HICs, or both?

Response: These are great points that highlight some of the unintentional HIC-biased statements made in the original text. We have adjusted this section of the introduction to try and emphasize (1) the pluralistic nature of what constitutes ‘impactful research’ which is traditionally and problematically defined by HIC groups and (2) the systematic barriers on multiple levels that have prevented LIC investigators and healthcare systems achieving epistemic and authorship equity.

Page 3, Lines 50-59: “…these are the geographic areas that have historically - and continue - to face significant systematic barriers to achieving equitable academic resources, institutional infrastructure, and economic development compared to high-income countries (HICs) (4, 5). Although there have been escalating calls for developing research capacity within LICs (6-8), the establishing of research priorities, definitions of what constitutes ‘meaningful’ research, and financial support for both primary research and research capacity building are dominated by HIC voices. Combined with the greater academic, institutional, and economic resources at the disposal of HIC investigators these inherent asymmetries in global health and infectious disease research end up perpetuating existing academic inequalities.”

**Comment 5:** Methods, page 4, line 65: Authors should define primary research (i.e. does this refer to primary data collection or is it synonymous with original research articles - excludes reviews/commentaries?)

Response: We have clarified the text to reflect that by primary research we are referring to primary data collection.

Page 5, Lines 83-84: “The focus of this study was primary research – defined as data collection, human subject research, or research using specimens from human subjects - located in one or more LICs…”

**Comment 6:** Methods, page 4, lines 66-68: Authors may consider elaborating on how their sample of these three journals represents infectious disease research more broadly. Are these the top 3 journals in this field? Are these most likely to publish research outside of HICs?

Response: Thank you for the comment. These journals were selected in response to the commitment made by the Infectious Disease Society for America (which oversees publication of the three journals analyzed) to be more representative of global academic research output. These are three high impact journals dedicated specifically to infectious disease topics that contribute significantly to establishing disease recommendations and standards of care. Following this study as a proof-of-concept, one strong consideration for further research we think would be very interesting includes an analysis between infectious disease journals with a wider range of impact factors and those considered most likely to publish research outside HICs and/or LMIC local and regional journals. This would be highly beneficial in potential identifying editorial trends and practices that promote research lead by LIC investigators. We have updated the text and discussion to include investigation into this question by other references.

Page 5, Lines 88-89: “…were selected due to the society’s record of publishing high-impact research, influence on the establishment of global standards for clinical care, and commitment to developing global health partnerships and furthering the international physician-scientist workforce.”

Page 16, Lines 360-365: “For example, in their analysis of authorship in sub-Saharan Africa, Hedt-Gauthier et al. found that over half (60.6%) of articles published in journals without impact factors had local authorship representation compared to only 4.8% of papers published in journals with impact factors over 10. A similar analysis within infectious disease literature and broader geographic focus would be of interest and relevant to our findings.”

**Comment 7:** Methods, page 4, lines 77-78: “results were then explored for associations with LICs based on title, keywords, or abstract text” was this done electronically (i.e. text parsing in statistical software) or manually? If manually, were there two reviewers, and how were disagreements resolved?

Response: We have added specifics regarding how the results were initially explored.

Page 5, Lines 98-99: “Results were then explored using an algorithmic text and keyword search in Microsoft Excel for associations with LICs based on title, keywords or abstract text.”

**Comment 8:** Methods: This journal may require authors to mention that ethics approval was waived/not required (at the editor's discretion).

Response: We apologize for the oversight and have updated both the manuscript text and disclosures section with ethics approval information.

Page 5, Lines 90-91: “This study was declared exempt from review by the Emory University Medicine Institutional Review Board.”

**Comment 9:** Results, page 6, line 121: Authors may consider briefly mentioning the formula for accuracy within the Methods section (i.e. I assumed formula was # articles with LIC affiliation/# articles; is this correct?)

Response: Thank you for pointing out that this requires clarification. We have included the formula used to determine accuracy during the audit process.

Page 7, Lines 145-146: “…data extraction demonstrated 96% accuracy (number of articles with correct study location and author affiliations/number of articles audited) in capturing…”

**Comment 10:** Results, page 7, line 134: Authors should define the acronym OFID on first use.

Response: We appreciate the reviewer pointing this out and have added the full journal title.

Page 8, Line 165: “There was no significant increase in the number of LIC articles by year when *Open Forum Infectious Diseases* was established in 2014.”

**Comment 11:** Results, page 8, line 164 (also applies to Methods, page 5, line 85): Did % first/last author increase/decrease over time among only the 73% of articles about countries that remained LIC over time? Generally, I am wondering how addition/subtraction of countries from certain years would have influenced authors' results re trends over time.

Response: We were similarly interested in whether the fluctuating between income statuses over the study time period would impact the result and why we chose to compare these two groups. Percentage of lead authors among LICs that remained low-income over the study period increased from 31% to 41% as reported in the text. For each year, the denominator used to calculate percentage of LIC authorship was the number of articles from countries defined as LIC that year. We therefore believe that using percentages still accurately captures the value of interest (whereas reporting number of articles, for example, would be impacted by fluctuations between income groups).

**Comment 12:** Discussion, page 11, lines 223-225: “This means that the increase in publications is attributable to non-LIC authorship” I think these findings are attributable “to a greater extent” to non-LIC authorship, because authors did also see increase in absolute numbers of publications with LIC first-/last- authorship (yet these increases were not proportional to increase in publications).

Response: We appreciate the attention to phrasing and have adjusted the text accordingly:

Page 12, Line 269: “This means that the increase in publications is mostly attributable to non-LIC authorship, the vast majority of which are authors from high-income countries.”

**Comment 13:** Discussion, page 11, lines 232-252, 311-324: See above comment re Introduction/Discussion from the perspective(s) of LICs. Authors may consider mentioning the role of LIC consultation and collaboration re top down and bottom up solutions. It would be, I think, an oversight for HIC researchers to assume they know what LIC want/need re building capacity and addressing barriers.

Response: A very good point we greatly appreciate the reviewer raising. We have added additional text to try and prevent any suggestion that capacity building should be HIC-centric.

Page 13, Lines 278-280: “Barriers to promoting LIC authorship remain rooted in the inequities that limit research-building capacity and vary greatly between different locations, partnership structures, and types of research. Any engagement in barrier identification and capacity building measures should be led by LIC investigators and communities. Such barriers include, but are not limited to, a paucity…”

**Comment 14:** Discussion, page 11, lines 253-267: I really liked the discussion of dual affiliation, and I think that exploration of dual affiliation is a strength of this study.

Response: Thank you, we appreciate hearing this discussion point was of interest and think it is one of the unique aspects of the analysis.

**Comment 15:** Discussion, page 13, lines 279-282: “These groups did have higher proportions of LIC authorship, although not statistically significant, when compared to HIV, tuberculosis and malaria, posing the question of the impact of international attention on local investigator development and authorship.” Authors may consider clarifying this sentence (I didn't understand its meaning).

Response: We have tried to clarify this part of the discussion. Our intention was to highlight that infectious diseases that traditionally have complex, multinational collaborative attention, for example the PEPFAR response to HIV/AIDS, had lower LIC authorship representation than some of the other disease categories. We acknowledge this is clearly a much more complicated topic that can be addressed by our analysis alone.

Page 15, Lines 337-341: “These groups did have higher proportions of LIC authorship, although not statistically significant, when compared to HIV, tuberculosis and malaria. This poses interesting questions around the impact of research topic on authorship equity including whether broadly endemic diseases like HIV and tuberculosis, which incur multinational research funding and collaborations, have differing complexities to the promotion of LIC investigator development compared to local disease outbreaks.”

**Reviewer 2 Comments**

**Comment 1:** …notwithstanding the irony of HIC first and senior authors on this paper (which implicitly criticizes the same patter in high-impact ID journals)

Response: We think this is a critical point to raise. The irony of our article’s authorship order is something we admittedly grappled with. As both reviewers have excellently pointed out, our analysis is impacted by the HIC-affiliation of both first and last authors. We have tried to rephrase any statements that suggest any HIC-centric thinking where able and remain open to further changes if editors and collaborators, particularly those with LMIC representation, have further recommendations.

**Comment 2:** Minor grammar and syntax errors. Throughout the manuscript, please ensure that commas are added prior to dependent clauses, particularly those that begin with adjectives and adverbs.

Response: We thank the reviewer for bringing this to our attention. The manuscript has been reviewed and the grammar/syntax errors that were found were corrected, including the following:

Page 3, Line 45: “Within low-income countries (LICs), almost…”

Page 7, Lines 141: “Among the 23,631 articles published in IDSA journals between 1998 and 2017, a total of 3233 were identified as being conducted within a LIC.”

Page 7, Line 143: After applying our inclusion and exclusion criteria, there were…”

Page 14, Lines 306: “But without a universal definition for claiming dual affiliation, there are a wide range of possibilities for what these affiliations mean.”

Page 14, Line 309: “Conversely, there is also…”

**Comment 3:** Introduction - Does “infectious diseases” need to be capitalized throughout this paper?

Response: The use of ‘infectious diseases’ in this article is in reference to the general category of diseases caused by pathogenic microbial agents. As referenced as a category and common noun they are not capitalized (versus, for example, reference to Infectious Diseases as a medical sub-specialty, which as a proper noun would be capitalized).

**Comment 4:** Methods - Search Strategy - Can the authors explain why the study period of 1998-2017 was chosen (given that it is now 2022)? What about this period (and, specifically, the 2017 cutoff) warrants study and why are more recent articles not included? One might argue that the global health movement has gained considerable steam in the past 5 years, so exclusion of articles beyond 2017 might bias the study.

Response: We agree the timeframe that was chose could contribute to some degree of bias and referenced this possibility in the limitations paragraph in the discussion. Further study into more recent publication trends and differences related to the COVID pandemic would be interesting to investigate. The cutoff of 2017 for our study was a pragmatic decision as data collection for this study was originally conducted in the middle of 2018. Prior to 1998, data was not reliably available through Scopus.

**Comment 5:** Inclusion/Exclusion - Line 85: Consider citing the World Bank County and Lending groups website in this section, as opposed to in the ensuing section, given that it is first mentioned here.

Response: We appreciate the reviewer pointing this out and have updated the citation.

Page 6, Line 97: “…conducted in one or more LICs as defined by the World Bank (16) according to year of publication.”

**Comment 6:** Results - Lines 125-6: “Errors in authorship affiliation missed the non-LIC affiliation for dual-affiliated authors although the LIC affiliation was accurate for these data.” I am very confused by this sentence. Can the authors re-phrase for clarity?

Response: Thank you for attention to this unclear phrasing. We have re-written the text to be more specific.

Page 7-8, Lines 149-150: “The error in identification of authorship affiliation for dual-affiliated authors was a result of not capturing their non-LIC affiliation.”

**Comment 7:** Authorship Trends - Line 134: The abbreviation “OFID” is used here, but should be defined in the search strategy section, where the full Journal title is used for the first time.

Response: We appreciate the reviewer pointing this out and have added the full journal title.

Page 8, Line 165: “There was no significant increase in the number of LIC articles by year when *Open Forum Infectious Diseases* was established in 2014.”

**Comment 8:** Lines 164-70: The authors raise an interesting question by (appropriately) mentioning that fewer countries were classified as LIC between the beginning and end of the study period. While the insinuation is present in the current text, I wonder whether the same trend of diminishing representative authorship would be present if the analysis included LMICs and LICs, rather than just LICs alone?

Response: This is a very good point. The duration of time studied and geographic range within our analysis and fluctuation of many countries into/out of LMIC or LIC classification made this difficult to parse out into meaningful data. However, a recent analysis by Dimitris et al. (<https://gh.bmj.com/content/6/1/e003758>) did find that LICs in particular, even when compared to LMICs and UMICs, were underrepresented in local authorship promotion in global health publications between 2000-2017. We have included this reference in the discussion at the recommendation of reviewer 1.

Page 12, Lines 256-259: “Large-scale metadata on global health research over the past 20 years indicates that proportions of non-HIC lead authors has increased over time, the majority of this trend was secondary to increases in authorship from upper-middle- and lower-middle- income countries, with LICs remaining underrepresented.”

**Comment 9:** Geographic Distribution - Figure 4: The maps add a nice touch and are easy to navigate. If appropriate, the authors should cite the program used to construct the maps in the Figure 4 heading.

Page 10, Lines 211-212: “…or last authorship. Maps were created using Plotly Chart Studio (Plotly Technologies Inc, released 2015). Abbreviations…”

Response: We appreciate the feedback. In discussion with a journal editor the map has been reformatted to ensure use of an open access and public domain map source and figure heading adjusted accordingly.

**Comment 10:** Lines 268-73: If the authors agree, I would consider a brief mention of overrepresentation of sub-Saharan (and, in particular, East African) research, which seems not to be isolated to Infectious Diseases studies (see: https://pubmed.ncbi.nlm.nih.gov/32959620/).

Response: Thank you for suggesting the above reference. We agree that it aligns with our findings and have incorporated it into the paragraph suggested by the reviewer.

Page 14, Lines 319-320: “Most publications in this analysis originated from the African continent and in particular East and West Africa. Similarly, prominent representation of research from sub-Saharan Africa has been reported in the global health literature (37). This may…”

References

(37) Ghani M, Hurrell R, Verceles AC, McCurdy MT, Papali A. Geographic, Subject, and Authorship Trends among LMIC-based Scientific Publications in High-impact Global Health and General Medicine Journals: A 30-Month Bibliometric Analysis. J Epidemiol Glob Health. 2021;11(1):92-7.

**Comment 11:** Lines 282-5: “Literature on Ebola had the lowest proportion of LIC authorship suggesting that emergency epidemics are a particularly complex research environment for LIC investigators with complicating factors that include urgency and politicized multiorganizational research collaborations.” Can the authors better explain what they mean by “urgency”? I would argue that emergency epidemics, such as Ebola Virus Disease, that create public health emergencies in LMIC settings markedly increase the work burden for local practitioners, leading to very little time/capacity to perform original research. Moreover, established, robust and well-funded research programs, most of which are based in HICs and which often collaborate directly with global health public bodies, such as the WHO, CDC and ECDC, usually have much greater capacity to ramp up scientific investigations on short notice.

Response: We agree it is worth clarifying the points being made in this section. By “urgency” we had been referencing the need for rapid creation and dissemination of research results to answer important questions about infectious pathogens like Ebola and SARS-CoV-2. We have adjusted the text to be more specific.

Page 15, Lines 341-345: “…literature on Ebola had the lowest proportion of LIC authorship suggesting that emergency infectious disease responses are a particularly complex research environment for LIC investigators with complicating factors that include increased clinical demands for local providers, the need for rapid dissemination of research findings, and politicized highly-resourced multiorganizational research collaborations (40).”

**Comment 12:** Lines 285-91: The authors raise an important point about LMIC representation during the COVID-19 pandemic. It may also be worthwhile to mention that many efforts are underway to address these inequalities through broad global collaborations. See, for example, https://www.ajtmh.org/view/journals/tpmd/104/3_Suppl/article-p1.xml.

Response: This is a good point to add to the discussion about research equity during the COVID-19 pandemic and we have added the suggested reference.

Page 16, Lines 353-354: “…inequities of healthcare provision in resource-limited settings (41, 42) and attention to addressing these disparities through collaborative global partnerships (43).”

References

(43) Dondorp AM, Papali AC, Schultz MJ. Recommendations for the Management of COVID-19 in Low- and Middle-Income Countries. Am J Trop Med Hyg. 2021.

**Comment 13:** Lines 297-8: Note that “phenomena” is plural, but the verb “contributes” denotes the singular.

Response: Thank you for bringing this grammatical discrepancy to our attention. We have adjusted the text to reflect phenomena as a plural noun.

Page 16, Line 368: “The same phenomena that contribute to episodic increase…”

Sincerely,

_________________________________________

**Corresponding Author:**

Chelsea Modlin, MD

PGY-5 Clinical/Research Fellow, Johns Hopkins University School of Medicine, Division of Infectious Diseases

Post-Doctoral Fellow, Hopkins-Oxford Global Infectious Disease Ethics (GLIDE) Collaborative

1830 E. Monument St, #450B

Baltimore, MD 21205

Phone: (802) 291-0004

Fax: (410) 835-9376

Email: cmodlin2@jhmi.edu

**Alternative Corresponding Author:**

Russell Kempker, MD, MSc

Associate Professor

Emory University School of Medicine, Division of Infectious Diseases

49 Jesse Hill Jr. Dr. SE

Atlanta, GA 30303

Phone: (404) 251-8701

Fax: (404) 880-9305

Email: rkempke@emory.edu
