# Supplementary material for: Authorship trends in infectious diseases society of America affiliated journal articles conducted in low-income countries, 1998–2018
Source: PLOS Glob Public Health. 2022 Jun 10;2(6):e0000275. doi: 10.1371/journal.pgph.0000275 (PMC10021251; doi:10.1371/journal.pgph.0000275)
Supplement: Attachment — Submitted filename: Response to Reviewers_FINAL.docx [file pgph.0000275.s004.docx]

April 20, 2022

Dear PLOS Global Public Health,

We have incorporated or responded to all of the suggestions made by the reviewers and included point-by-point responses below. All page numbers refer to the revised manuscript file with tracked changes. Thank you for the opportunity to revise and resubmit our manuscript.

**Reviewer 2 Comments**

**Comment 1:** In the limitations, please briefly explain why the cutoff date of 2017 was chosen and the impact that this choice could have had on the analysis.

Response: This work/analysis was initiated in 2018 and hence we utilized the most up to date dataset available at that time. To provide clarification on this point, we have added the below information to the limitations paragraph of the discussion.

Page 16, Lines 341-343: “…Ebola epidemic. Additionally, the preliminary analysis for this paper was conducted in 2018 and the end date of 2017 does not investigate more recent authorship trends. Due to…”

**Comment 2:** "low-income setting" is used in the Introduction, whereas "resource-limited settings" is used in the Discussion. While the two terms often are used synonymously, they can mean different things to different people. Since the study focus is on LICs, consistency in terminology would be beneficial.

Response: Thank you for pointing this out as it is an important distinction to make. We have edited the discussion to reflect terminology used throughout the rest of the paper. After making this change, the term low-income setting or low-income country is used throughout the manuscript.

Page 16, Lines 322: “…spotlight on inequities of healthcare provision in low-income settings (41, 42) and attention…”

**Reviewer 3 Comments**

**Comment 1:** The manuscript's insights may be weakened by some methodological concerns…The authors' repository choice does not help extrapolate their findings to the original target population - LIC infectious disease original research because the authors chose the Infectious Disease Society of America-affiliated journals… First, we can reasonably expect that the journal holds “high-standards” and therefore has a low acceptance rate. We know from previous research that “high-impact factor” journals are less likely to publish research from low- and middle-income country affiliated researchers even when this research is methodologically sound…Next, the journals included are run by the “Infectious Disease Society of **America**,” – we would be misguided to assume that this **American** based journal is a representative of LIC infectious diseases research…The limited repository restricts the authors’ findings to the Infectious Disease Society of America and I believe it will be a shame to limit this study’s impact this way.

Response: We appreciate this response and agree with the reviewer that the selection of journals used for the analysis is a limitation. As such, we have expanded on the discussion of this limitation and have adjusted the text in several places to narrow the applicability of the results. We hope these changes better reflect our original research question and objective as a proof-of-concept study investigating a limited number of journals across an extended period of time given previous analyses have been limited by disease process, geographic region, and/or by shorter (typically 1-5 year) timeframes. We do feel that our work provides valuable insights regarding LIC-affiliated authorship contributions to global academic knowledge networks but do not wish to purport that our work is representative of LIC infectious disease research as a whole.

Page 2, Line 39: “…yet authorship trends in a small subset of these publications demonstrate…”

Page 11, Line 210: “We analyzed a subset of primary infectious disease research based in LICs or involving LIC participants published between 1998 and 2017.”

Page 11, Line 214-216: “Our findings indicate more efforts are needed to enhance LIC authorship equity within high-impact infectious disease journals.”

Page 12, Line 235: “Our study demonstrates a similar distribution of research from LICs within a subset of the infectious disease literature.”

Page 16, Lines 326-329: “Only 5.5% of articles that were both published in the three journals analyzed and met inclusion criteria were conducted in LIC settings. These journals are not wholly representative of infectious disease research in LICs more generally. The focus on few journals for our analysis acknowledges there is a degree of publication bias within our results. These journals represent only a fraction…”

**Comment 2:** There are other repositories that will give a less restricted view of infectious diseases research: the World Health Organization’s Global Health Index, PubMed, Embase, Web of Science, and Google Scholar. We recommend the authors develop a comprehensive search strategy to cover the key words: infectious diseases and LIC. Then, they can download the results as bibliometric metadata (ex: CSV, bibtex, etc). They would then deduplicate the articles and upload them in a bibliometric software like Bibexcel, CiteSpace, Histcite, Pajek, Publish or Perish, R, Scholarometer, or VOS viewer for analysis. Some of these software can perform first and last author analysis automatically.

Response: Thank you for the detailed attention and suggestion. We agree that looking at a broader range of journals and impact factors would be an interesting analysis but do feel it falls outside of the scope of this particular manuscript described in the response to comment #1. We hope that the changes made above are satisfactory for clarification. This project was initiated in 2018 and its methods involved a time-intensive manual clean-up of the data set generated from Scopus output and detailed review of articles to confirm data and validate the search criteria. In an effort to maximize its relevance and minimize time between the data period and dissemination we hope it can be appreciated that recollecting and reanalyzing the majority of data is unfortunately not feasible in a timely fashion. A separate paper investigating prominent endemic infectious diseases (HIV/AIDS in particular) with similar methodology to what the reviewer is suggesting, including a wider data repository and range of journal impact factors, is currently in process by some members of our research group.

**Comment 3:** It is critical that the authors differentiate between LIC authors and LIC-affiliated authors. Based on the authors methodology section, it seems more appropriate to talk of the LIC-affiliated authors.

Response: This is an important distinction and we are grateful it has been brought to our attention. We have adjusted the text to use ‘LIC-affiliated author in reference to our analysis but left the term as ‘LIC author/authorship’ when referencing other publications and in the general discussion given potential differences in methodology or definitions. The suggested changes have been made in the following locations:

Page 2, Lines 32-40

Page 6, Line 106

Page 7, Line 120

Page 8, Lines 152, 154

Page 9, Figure 2 caption, Lines 161-164

Page 9, Figure 3 caption, Line 166

Page 9, Line 174

Page 10, Line 186

Page 10, Figure 4 caption, Line 197

Page 11, Line 207, 215

Page 12, Lines 244, 245

Page 14, Lines 298

Page 15, Lines 310, 316

Page 17, Line 352

We have additionally added one citation based on a publication released since the original manuscript was submitted.

Page 11, Lines 218-219: High-impact research in LICs has historically been under-represented in the medical literature (18, 19) despite social and ethical arguments for research to be based on relevant global disease burden (20).

Sincerely,

_________________________________________

Chelsea Modlin, MD

PGY-5 Clinical/Research Fellow, Johns Hopkins University School of Medicine, Division of Infectious Diseases

Post-Doctoral Fellow, Hopkins-Oxford Global Infectious Disease Ethics (GLIDE) Collaborative

Russell Kempker, MD, MSc

Associate Professor

Emory University School of Medicine, Division of Infectious Diseases
